# The Effect of Complex Modification on the Impedance of Cement Matrices

**DOI:** 10.3390/ma14030557

**Published:** 2021-01-24

**Authors:** Grigory Yakovlev, Černý Vít, Irina Polyanskikh, Anastasiya Gordina, Igor Pudov, Alexander Gumenyuk, Olga Smirnova

**Affiliations:** 1 Department of Geotechnical Engineering and Building Materials, Kalashnikov Izhevsk State Technical University, Studencheskaya Str. 7, 426009 Izhevsk, Russia; gyakov@istu.ru (G.Y.); irina_maeva@mail.ru (I.P.); afspirit@rambler.ru (A.G.); pudovia@yandex.ru (I.P.); aleksandrgumenyuk2017@yandex.ru (A.G.); 2 Faculty of Civil Engineering, Brno University of Technology, Vevěrí 95, CZ-6S1200 Brno, Czech Republic; cerny.v@fse.vutbr.cz; 3 Department of Constructing Mining Enterprises and Underground Structures, Saint-Petersburg Mining University, 199106 Saint-Petersburg, Russia

**Keywords:** impedance, cement binders, morphology, structure, multiwall nanotubes, complex modification

## Abstract

The research results presented in this article were obtained by joint scientific research on creatingcement materials with reduced impedance. It is known that functional additives added to impart electrically conductive properties have a negative impact on physical and mechanical characteristics of the material. This study suggests using the multiwall carbon nanotubes in the amount of 7% from binder mass as a functional additive. The results obtained prove that the addition of this amount of the modifier does not lead to a significant decrease of strength characteristics. Calcium nitrate in the amount of 1–7% was added in order to level the strength loss and to ensure the effective stable electrical conductivity. The multifunctionality of using this salt has been proven, which is manifested in the anti-frost and anticorrosive effects as well in enhancement of electrical conductivity. The optimal composition of the additive with 7% of carbon nanotubes and 3% of calcium nitrate ensures a reduced electrical impedance of cement matrix. The electrical conductivity was 2440 Ohm, while the decrease of strength properties was within 10% in comparison tothe control sample. The nature of changes in the microstructure were studied to determine the influence of complex modifications that showed significant changes in the morphology of the hydration products. The optimum electrical characteristics of cementitious materials are provided due to the uniform distribution of carbon nanotubes and the formation of a network of interconnected micropores filled with the solution of calcium nitrate that provides additional and stable electrical conductivity over time.

## 1. Introduction

Research on technological methods of imparting electrical conductivity to cementitious concretes is carried out to expand the functional properties of conventional building materials [1,2]. Today, electrically conductive cement materials have become widespread in the construction industry and have been actively studied over the past two decades [3,4,5].

Electrically conductive cement materials are used to prevent icing and snow accumulation in the structures of road surfaces, automobile bridges, parking lots, sidewalks and runways due to heat emission [6,7]. Electrically conductive cement materials are used as cathodic protection systems for steel reinforcement in reinforced concrete [4] as well as self-diagnosing building systems for monitoring the stress–strain behavior of structures in real time [2].

In addition, electrically conductive cement materials are used in the production of antistatic floors and as electromagnetic reflectors to protect against electromagnetic interference [8]. At the same time, electrically conductive cement concrete also has sufficient potential for use in grounding systems [9] that are of decisive importance for buildings with a continuous presence of people (residential buildings, offices, industrial premises) as well as power facilities.

The production of functional cement concrete is possible by mixing the traditional components (binder, coarse and fine aggregates, water) with electrically conductive components providing stable electrical properties [10].

Today, composite materials based on Portland cement are widely used [3,4,11]. Furthermore, this type of binder prevails in the areas of construction whichrequire taking into account or using electrical properties of material.

As noted in studies [5,12], the cement matrix based on Portland cement is a combination of hydration products with unreacted clinker minerals, which are thermodynamically unstable compounds with defective structures. The authors of the paper [13] determined the change in entropy in the reactions of the formation of calcium silicates, aluminates and aluminoferrites. This made it possible to state that the sum of the entropies of the initial oxides is much less than the entropy of the clinker minerals themselves.

In view of this, it can be said that the minerals of Portland cement clinker have a disordered crystal structure with vacant sites in the lattice points and also contain a variety of ions with significantly weaker bonds located in the interstices. The presented features of structure of cement minerals influence the physical and chemical properties of the material including the ability to transmit electrical current. The joint motion of the thermal and electric field can lead to the fact that one field can become a current carrier, which determines predominantly ionic conductivity of cement matrices. The value of this type of electrical conductivity depends on the degree of orderliness of ions in the crystal lattice [13].

Factors such as the water-to-cement ratio [14], the volume of pore space [5] and the amount of adsorbed moisture in the mineral structure [11] also affect the electrical conductivity of cement matrices, along with the degree of structure crystallization.

In particular, the relationship between the conductivity of cement gel as a colloidal system and the presence of moisture in it was stated in studies [2,5,11]. This dependence determines that over time the cement gel is permeated with crystalline hydration products. As a result, a decrease of the amount of free ions and an increase of the crystallization degree occurs. This, in turn, reduces the electrical conductivity of the material and increases its electrical resistivity [14].

The effect of the crystallization degree on the increase of resistivity was also confirmed by studies of low-basic calcium hydrosilicates [4,5,11] and it was found that calcium hydrosilicates with the basicity close to one have the highest crystallization degree. Calcium hydrosilicates with the degree of basicity from 0.8 to 1.3 have an increased electrical resistivity according to the conclusions of paper [15].

Traditional cement materials based on Portland cement have increased resistivity, the values of which vary from 6.54 to 11 kOhm [11]. In this case, the addition of conductive carbon-containing additives (for example, graphite, soot, etc.) is the most common way to increase the conductivity of cement composite [2,3,10,16].

Along with this, it is necessary to take into account that the amount of conductive components should not exceed certain limits to provide the percolation effect for improving the electrical properties of cement material [17,18] as well as to avoid the negative impact on physical and mechanical properties of concrete [14]. It is known that highly dispersed additives are used to increase mechanical properties of cement-based composites. However, the proper amount of the additives varies in the range 0.001–0.01% [19,20,21,22]. In the case of conductive materials, such an amount of modifiers is not suitable due to different mechanisms that provide the conductivity. There, we stated that the increase of dispersed additives provides the required conductivity [23,24], but decreases the mechanical properties of matrices, especially the compressive strength [25].

According to paper [26], the use of soot and multiwall carbon nanotubes promotes a distribution of electrically conductive components in material structure that provides stable electrical characteristics without worsening the physical and mechanical properties [1,5].

The mechanism of reduction of electrical resistance of a material is considered from the standpoint of optimal structure formation in which the electrically conductive particles forms cluster bonds [27] contributing to the appearance of the effect of charge transfer over long distances [28]. In view of this it can be argued on the basis of research [16] that the achievement of a consistently high electrical conductivity is possible with a uniform distribution of electrically conductive components in material structure.

However, there are factors that determine the instability of electrical properties. They are associated with increased humidity due to operating conditions, with the blocking of electrically conductive ions by hydration products as well as with the issues of ensuring high initial strength and density of materials based on cement binders. This significantly limits the development of an efficient electrically conductive material due to the use of one functional modifier [29].

A decrease of crystallization degree of structure of cement matrix is possible due to the use of various salts, in particular calcium nitrate, as shown by laboratory studies [30] and the experience of SINTEF Company [31].

Today, calcium nitrate is used as an accelerator of the setting time of hardening cement paste and an inhibitor of electrochemical corrosion. The use of calcium nitrate solves the problem of decreasing strength, both at the initial hardening stage and when gaining strength within the project time. It should also be noted that calcium nitrate exclusively effects the morphology of secondary crystalline hydrates of the cement matrix [30,32]. Based on the above, it can be assumed that a complex effect expressed in a consistently low specific electrical resistance and increased strength properties of the material can be obtained with the combined use of calcium nitrate and electrically conductive components.

The aim of the paper is to determine the effect of complex modification including multiwall carbon nanotubes and sodium nitrate on electrical properties of materials based on Portland cement.

## 2. Materials and Methods

Portland cement CEM I 42.5 N was used as the main binder. The chemical and mineralogical composition of the clinker is represented by the following minerals, %: tricalcium silicate C_3_S-64.6, dicalcium silicate C_2_S-10.7, tricalcium aluminate C_3_А-7.0, tetracalciumaluminoferrite C_4_AF-14.7, MgO-1.4. Chemical and mineralogical compositions of cement have been confirmed by manufacturer’s product bulletin provided by Eurocement Group. 

Polyfractional natural quartz sand conforming to EN 196-1 was used as fine aggregate. The sand grains were predominantly rounded. The silica dioxide amount in sand was more than 98%.

Control mortar (CSM) had the ratio of cement-to-sand equal to 1:3 by mass. Modification of the mortar was done by adding Fulvek 100 multiwall carbon nanotubes in the amount of 7% by cement mass (manufactured on the basis of Graphistrength TM Masterbatch CW 2-45 ArkemaCo, Kolombe, France). The particular amount of multiwall carbon nanotubes had been defined by previous studies [33,34]. Fresh mortars of the standard consistency were used for the manufacture of sample beams with dimensions of 40 mm × 40 mm × 160 mm.

Carbon nanotubes (CNTs) were added into 150 mLof water to obtain an aqueous modifying suspension, which was then treated with a HielscherUP200 ultrasonic homogenizer (Hielscher, Tetlow, Germany) for 5 min at 150 W, the frequency of 26 kHz and the maximum amplitude of 70 μm. Additionally, calcium nitrate in the amount from 1% to 7% by cement mass was added into the mixing water with the step of 1%.

At the age of 7, 14 and 28 daysthe flexural strength and compressive strength tests were performed for control and modified samples by using standard beam-shaped samples and hydraulic press. Coefficient of variation was 13.5%.

**Figure 1:**
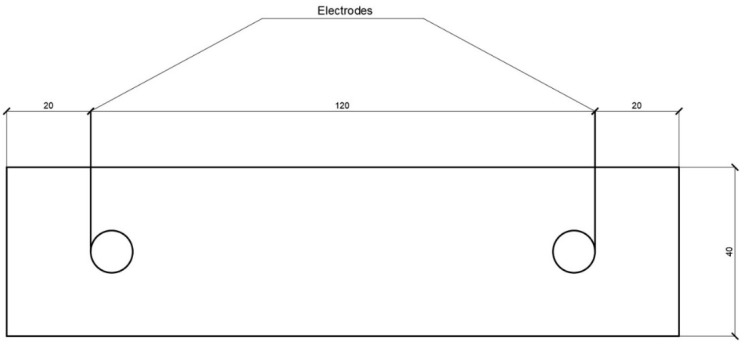
Electrical conductivity measuring diagram.

The measuring diagram shown in Figure 1 was used to determine the impedance.

The MNIPI E7-20 device was used to determine changes in the electrical conductivity of layers and resistivity. The operating principle of the device is based on the voltmeter-ammeter method. The voltage of the operating frequency from the generator is fed through the measured object to the converter that forms two sinusoidal voltages (proportional to the current flowing through the object and proportional to the voltage on the object). Voltages are converted into digital form.

IR Fourier spectrometer (Shimadzu, Kyoto, Japan) “IRAffinity-1” in the frequency range 4000 ÷ 400 cm^−1^ in transmitted light was used to analyze the samples by infrared spectroscopy.

The analysis of the microstructure of the samples was conducted using scanning electron microscopes (Thermo Fisher Scientific, Waltham, MA, USA) ThermoFisher Scientific Quattro S. The shooting was carried out in the low vacuum mode at 20 kV, without deposition, at a pressure of 50 Pa.

## 3. Results

**Figure 2:**
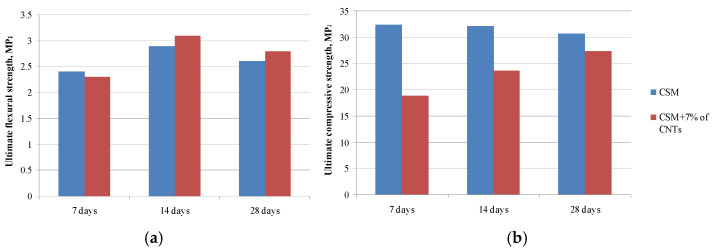
Strength characteristics in different periods of hardening: Flexural strength (**a**), compressive strength (**b**).

The physical and mechanical properties of the compositions were determined to assess the effect of modifying additives. The flexural strength at the age of 28 days of the modified sample exceeds the corresponding parameter of the control sample by 7% and the compressive strength decreases slightly within 10% (Figure 2). At the age of 28 days compressive and flexural strength changes slightly due to the fact that the role of multiwalled carbon nanotubes in this case only as a filler.

**Figure 3:**
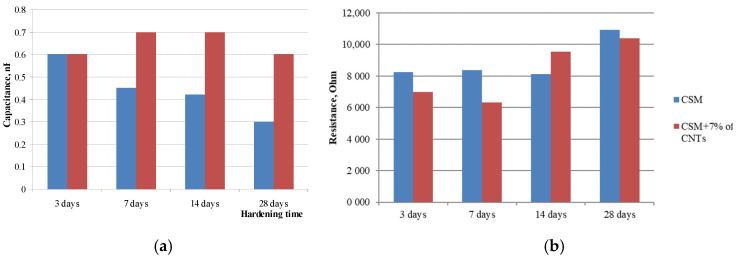
Changes of electrical characteristics of control sample (CSM) and sample modified with 7% of carbon nanotubes (CNTs): electrical capacity (**a**), resistance (**b**).

**Figure 4:**
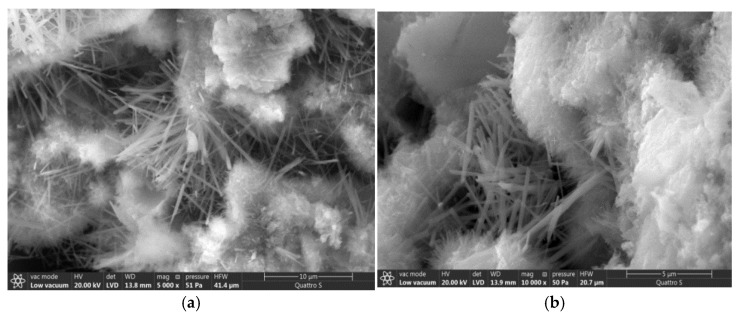
The microstructure of the matrix modified with 7% of carbon nanotubes at various magnifications: 5000 (**a**), 10,000 (**b**).

Determination of electrical characteristics and comparison of control and modified samples showed that the use of the optimal amount of carbon nanotubes in the amount of 7% by cement mass has a positive effect on electrical capacity and resistance. Thus, the electrical capacity of the modified sample exceeds the electrical capacity of the control one by 2 times, while the resistance of the modified sample at the age of 28 days decreases up to 12% (Figure 3). The addition of multiwall carbon nanotubes leads to the formation of a volumetric spatial grid of nanostructures and hydration products that one can see below in the micrographs. This grid provides for the free movement of charged particles, probably due to the occurrence of a tunnel effect similar to the percolation effect [35] that occurs in polymer matrices, which is confirmed by the results of determining the electrical capacity: the control sample “accumulates” the charge, while the addition of additives can significantly reduce the electrical capacity (Figure 3a). However, over time the positive effect of addition of multiwall carbon nanotubes decreases due to the formation of a volumetric structural framework including calcium hydroxides and calcium hydrosilicates (Figure 3b and Figure 4).

The changes of electrical characteristics over time can be justified by the process of structure formation and the formation of the matrix framework that is gradually filled with calcium hydrosilicates. The volume of the liquid phase is significantly reduced contributing to the resistance increase. Multiwall carbon nanotubes also contribute to the processes of structure formation. During hardening they are gradually covered with hydration products that block charge transfer which leads to an insignificant difference in the resistance values of the control and modified samples at the age of 28 days.

IR spectral analysis and microstructure analysis were performed to determine the mechanism of the influence of carbon particles on the structure and composition of cement matrix.

**Figure 5:**
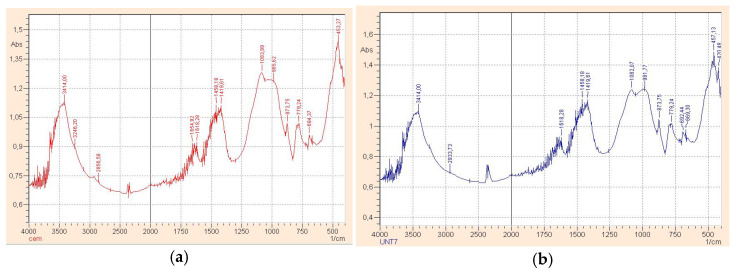
Results of IR-spectral analysis of samples: control composition (**a**), modified composition (**b**).

**Figure 6:**
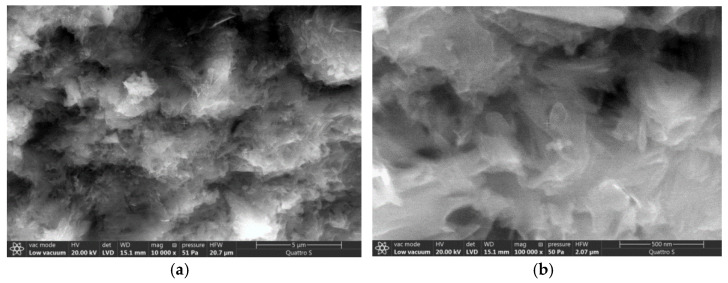
Microstructure of cement matrix of control sample: (**a**) at 10,000-fold magnification, (**b**) at 100,000-fold magnification.

Several characteristic groups should be distinguished when comparing the obtained spectra in Figure 5 and Table 1. A ratio of components in the cement matrix might be identified due to the fact of modulation in the relative intensity of absorption lines. The addition of carbon nanotubes leads toa change of conditions for the formation of the solid phase since a change of the ratio of the intensities of spectral lines caused by the presence of stretching vibrations of hydroxyl and silicate groups indicates this (Figure 5b). Amorphous structures forming the dense conglomerates prevail in the structure of the control sample at the age of 28 days (Figure 6).

The morphology of hydration products is changed byadding the carbon modifiers; namely crystalline hydration products, including calcium hydroxide, are formed in much larger amounts (Figure 6). The crystalline structure of the hardened cement matrix improves the conductivity of charged particles and the accumulation of charge occurs in a smaller volume.

**Figure 7:**
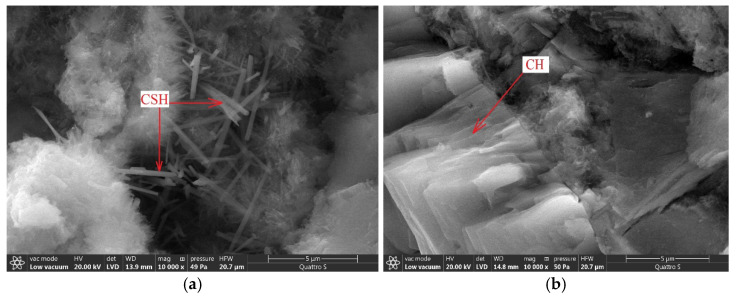
Morphological features of hydration products at 10,000-fold magnification: (**a**) sample with 7% of carbon nanotubes (**b**) sample with 7% of carbon nanotubes and 3% of calcium nitrate.

The functional role of carbon nanotubes in the cement matrix can be assumed based on the localization of hydration products (Figure 6). The functional role of carbon nanotubes is expressed in the formation of uniformly distributed crystallization centers that directionally structure the hydration products (Figure 6 and Figure 7a). In addition, the carbon nanotubes act as a framework element embedded in the amorphous gel-like structure of the cement matrix to ensure the directional movement of charged particles. The decision to further modify this composition with calcium nitrate was made taking into account the effect of blocking carbon nanotubes with hydration products over time and compensating the strength loss.

**Figure 8:**
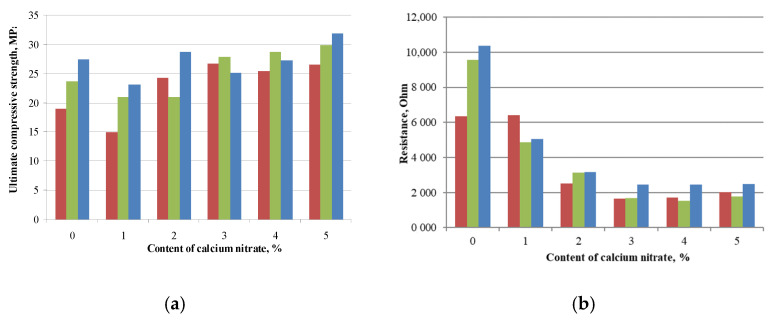
Influence of calcium nitrate amount on characteristics of sample with 7% of carbon nanotubes: ultimate compressive strength (**a**), electrical resistance (**b**).

It is known [36] that calcium nitrate is a hardening accelerator of cement due to creation of an excess of calcium cations in liquid phase of cement paste. The effect of the additive is expressed in an increase of compressive strength and in an additional decrease of electrical resistance as shown in Figure 8.

Thus, calcium nitrate, depending on the amount, added provides the increase of compressive strength up to 20–30% as shown in Figure 8a. At the same time the decrease of electrical resistance of sample with increasing the calcium nitrate amount was noted. It can be explained by the formation of an electrolyte solution in the liquid phase of cement matrix pores that ensures the electrical conductivity increase. The optimal composition of additive with 7% of carbon nanotubes and 3% of calcium nitrate provides a simultaneous sufficient strength (flexural strength increase by 50% and compressive strength decreases by 8%) and a decrease of electrical resistance (by 75%) without formation of efflorescence on sample surface. Electricalresistance obtained for samples with combined additive (multiwalled carbon nanotubes and calcium nitrate) is correlated with published research data [37,38]. The microstructure of the modified cement matrix was studied to determine the possible mechanism providing the electrical conductivity was sufficient.

It is known [39] that the increase of structure density leads to the increase of electrical conductivity of material. The increase of material density with the addition of multilayer carbon nanotubes should be noted according to Figure 6 and Figure 7a. In this case, the forming crystal hydrates are transformed from needle-like crystals (Figure 7a) into dense lamellar structure (Figure 7b) with addition of 7% carbon nanotubes and 3% calcium nitrate.

**Figure 9:**
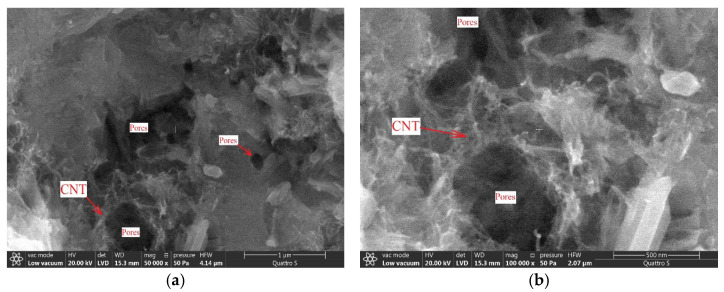
Pores formed with nanotube conglomerates: (**a**) at 50,000-fold magnification, (**b**) at 100,000-fold magnification.

Uniform distribution of multiwall carbon nanotubes in the material (Figure 9) provides the structuring of hydration products in cement matrix.

The creation of a network of connected micropores that are filled with salt solutions is proposed in paper [40] in order to increase the electrical conductivity of cement matrix. Nanopores of sizes from 100 to 400 nm were found in the structure of cement matrix when 3% of calcium nitrate was added (Figure 9a). Carbon nanotubes are involved in the formation of such pores since they create a kind of wall in the channels (Figure 9b).

**Figure 10:**
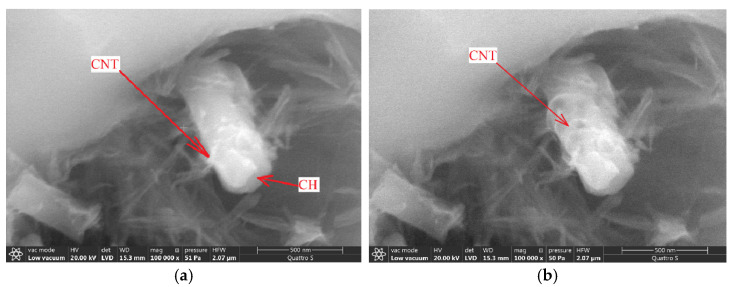
Calcium hydroxide with nanotubes: morphology at the initial moment of study (**a**), changes in morphology under the influence of an electron beam (**b**).

The role of carbon nanotubes in the formation of the structure of hydration products can be defined by analysis of the microstructure of a calcium hydroxide crystal with carbon nanotubes (Figure 10). Under the influence of an electron beam, due to dehydration of calcium hydroxide, nanofibers from the structure of a block of calcium hydroxide plates appear on its surface. Carbon nanotubes located in the structure of calcium hydroxide plates appear on the surface of plates block after exposure to an electron beam due to dehydration of calcium hydroxide.

**Figure 11:**
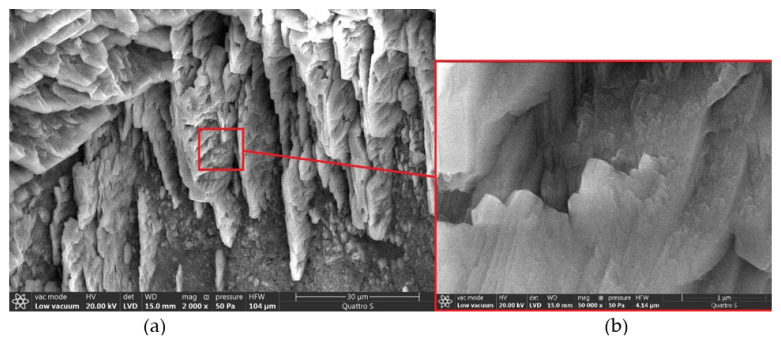
Microstructure of calcium hydroxide on the pore surface in the modified cement matrix of optimal composition at different magnifications: (**a**) 2000, (**b**) 50,000.

The noted structural features of the modified cement matrix make it possible to explain the increase of its electrical conductivity by the presence of uniformly distributed carbon nanotubes in hydration products that provide a decrease of electrical resistance. At the same time the electrical conductivity of the cement matrix continues to decrease in period from 3 to 28 days due to the formation of new hydration products that screen the surface of nanotubes as well as due to the transition of calcium nitrate from the liquid phase to the structure of secondary hydration products. Secondary hydration products are formed on free surface of pores with the formation of lamellar aggregates including calcium hydroxide (Figure 11).

The morphology of the cement matrix with the combined addition of carbon nanotubes and calcium nitrate is compacted with the formation of lamellar crystalline hydrates. The modified structure of the cement matrix provides the conductivity of charged particles and a decrease of electrical resistance of the material while the strength characteristics increase in comparison to the control composition.

## 4. Conclusions

The following conclusions can be drawn by analyzing the results obtained:

- the slight decrease of compressive strength is observed when using multilayer carbon nanotubes in the amount of 7% as an impedance-reducing modifier; at the same time the decrease of electrical resistance of samples was 12% at the age of 28 days of hardening compared to the control additive-free sample;
- the increase of the electrical conductivity of the composition with 7% of carbon nanotubes should be noted in the period of structure formation from 3 to 28 days due to changes in the morphology of hydration products. The influence of microstructure features on the electrical conductivity of the cement matrix should be indicated since amorphous hydration products in the control sample determine the unsatisfactory electrical properties;
- the positive effect of the complex modification with calcium nitrate and carbon nanotubes has been determined. It consists of sufficient strength characteristics while reducing the electrical resistance. The composition with the content of 7% carbon nanotubes in combination with 3% calcium nitrate ensures the compressive strength of 28 MPa and the electrical conductivity of 2440 Ohm.

## Figures and Tables

**Table 1:** Information of positions from FTIR.

Wavenumber (cm^−1^)	FTIR Peak Origin
3000–3500	Hydroxyl group
1600–1650	Behind vibration of water
900–1100, 680–780	Si-O-Si stretching in calcium hydrosilicates
1400–1450, 873, 75	Carbonate group

## Data Availability

Data is contained within the article material.
